# A System of Surgery

**Published:** 1845-07

**Authors:** 


A SYSTEM OF SURGERY. By *J. M. Chelius,* Doctor in Medicine and Surgery, Public Professor of General and Ophthalmic Surgery, Director of the Chirurgical and Ophthalmic Clinic in the University of Heidelberg, &c. &c. Translated from the German, and accompanied with additional Notes and Observations. By *John F. South,* Surgeon to St. Thomas's Hospital. 8vo. Parts I. & II., pp. 208. Renshaw, 1845.

THIS work has long been the chief text-book on surgery in the principal schools of Germany, and the publication of five editions of it in the original and of translations into no less than eight foreign languages, show the high estimation in which it is held. As a systematic work on surgery it has merits of a high order. It is methodical and concise—and on the whole clear and accurate. The most necessary information is conveyed in the shortest and simplest form. Minor details and fruitless speculations are avoided. It is, in fact essentially a practical book. This work was first published nearly twenty years ago, and its solid and permanent reputation has no doubt led Mr. South to undertake the present translation of the latest edition of it, which, we are informed, is still passing through the press in Germany. We should have felt at a loss to select any one better qualified for the task than the Translator of Otto's Compendium of

Human and Comparative Pathological Anatomy,—a surgeon to a large hospital, whose industry and opportunities have enabled him to keep pace with the improvements of his time. In a work like the one under review, which is intended chiefly as a class-book for students, however excellent the original may be, some additions and corrections are required in a translation. The principles of surgery must of course equally apply in all countries and climates; still, the character of the practice in Germany, Great Britain and France differs to such an extent, that the directions suited for the practitioners in one country would not altogether meet the views of those in another. The necessity for these corrections, if frequent, interferes, however, with the utility of the work, and to a certain extent renders the translation unsuited to be an elementary book for students, who should indeed be informed of the latest improvements in surgery, but whose attention ought not to be distracted by critical remarks and cautions against adopting the views recommended in the text. The length of Mr. South's notes will perhaps surprise our readers. In the first thirty pages, they bear a proportion to the text of about two-thirds to one-third, consisting chiefly of compilations from the works of the most recent writers on inflammation. Now, if so much additional matter were required to adapt the work for English readers, we think that the indefatigable translator might, without much increase of labour, have himself written a systematic book on surgery, which would be more useful and more acceptable to the rising generation of surgeons than the work he has translated and edited. We think too, that these very copious notes affect the character of the original. They clearly indicate its poverty and insufficiency, and if they render the translation more comprehensive than the original, they likewise render it less concise and didactic. It is time, however, that our readers should have an opportunity of forming their own opinion on the merits of this work, and of the translator's labours.

After a brief introduction, giving a definition of Surgery, and exhibiting its relation to the healing art in general, the author gives "the following division for the setting forth of surgical diseases which, if it be open to many objections, is, however, an arrangement of diseases according to their internal and actual agreement:—

"I. Division.—*Of inflammation.*

1. *Of inflammation in general.*
2. *Of some peculiar kinds of inflammation.*
   *a.* Of erysipelas; *b.* Of burns; *c.* Of frost-bite; *d.* Of boils; *e.* Of carbuncle.
3. *Of inflammation in some special organs.*
   *a.* Of inflammation of the tonsils; *b.* Of the parotid gland; *c.* Of the breasts; *d.* Of the urethra; *e.* Of the testicle; *f.* Of the muscles of the loins; *g.* Of the nail-joints; *h.* of the joints, viz.
   a. of the synovial membrane; b. of the cartilages; c. of the joint-ends of the bones, viz., *aa.* in the hip-joint; *bb.* in the shoulder-joint; *cc.* in the knee-joint; and so on.

II. Division.—*Diseases which consist in a disturbance of physical connexion.*

1. *Fresh solutions of continuity.*
   A. Wounds; B. Fractures.

II. *Old solutions.*

A. Which do not suppurate, viz.

*a.* False joints; *b.* Hare-lip; *c.* Cleft in the soft palate; *d.* Old rupture of the female perineum.

B. Which do suppurate, viz.

i. *Ulcers.*

1. In general.
2. In particular.

*a.* Atonic; *b.* Scorbutic; *c.* Scrofulous; *d.* Gouty; *e.* Impetiginous; *f.* Venereal; *g.* Bony ulcers or caries.

ii. *Fistulas.*

*a.* Salivary fistula; *b.* Biliary fistula; *c.* Fæcal fistula and artificial anus; *d.* Anal fistula; *e.* Urinary fistula.

III. *Solutions of continuity by changed position of parts.*

1. Dislocations; 2. Ruptures; 3. Prolapses; 4. Distortions.

IV. *Solutions of continuity by unnatural distension.*

1. In the arteries, aneurisms; 2. In the veins, varices; 3. In the capillary vascular system, teleangiectasis.

### III. DIVISION.—*Diseases dependent on the unnatural adhesion of parts.*

1. Anchylosis of the joint-ends of bones; 2. Growing together and narrowing of the aperture of the nostils: 3. Unnatural adhesion of the tongue; 4. Adhesion of the gums to the cheeks; 5. Narrowing of the œsophagus; 6. Closing and narrowing of the rectum; 7. Growing together and narrowing of the prepuce; 8. Narrowing and closing of the urethra; 9. Closing and narrowing of the vagina and of the mouth of the womb.

### IV. DIVISION.—*Foreign bodies.*

1. *Foreign bodies introduced externally into our organism.*

*a.* into the nose; *b.* into the mouth; *c.* into the gullet and intestinal canal; *d.* into the wind-pipe.

2. *Foreign bodies formed in our organism by the retention of natural products.*

A. Retentions in their proper cavities and receptacles.

*a.* Ranula; *b.* Retention of urine; *c.* Retention of the fœtus in the womb or in the cavity of the belly (Cæsarean operation, section of the pubic symphysis, section of the belly).

B. Extravasation external to the proper cavities or receptacles.

*a.* Blood swellings on the heads of new-born children; *b.* Hæmatocele; *c.* Collections of blood in joints.

3. *Foreign bodies resulting from the accumulation of unnatural secreted fluids.*

*a.* Lymphatic swellings; *b.* Dropsy of joints; *c.* Dropsy of the bursæ mucosæ; *d.* Water in the head, spina bifida; *e.* Water in the chest and empyema; *f.* Dropsy of the pericardium; *g.* Dropsy of the belly; *h.* Dropsy of the ovary; *i.* Hydrocele.

4. *Foreign bodies produced from the concretion of secreted fluids.*

### V. DIVISION.—*Diseases which consist in the degeneration of organic parts, or in the production of new structures.*

1. Enlargement of the tongue; 2. Bronchocele; 3. Enlarged clitoris; 4. Warts; 5. Bunions; 6. Horny growths; 7. Bony growths; 8. Fungus of the dura mater; 9. Fatty swellings; 10. Encysted swellings; 11. Cartilaginous bodies in joints; 12. Sarcoma; 13. Medullary fungus; 14. Polyps; 15. Cancer.

VI. Division.—*Loss of organic parts.*

1. *Organic replacement of already lost parts,* especially of the face, according to the Tagliacozian and Indian methods.
2. *Mechanical replacement:* Application of artificial limbs, and so on.

VII. Division.—*Superfluity of organic parts.*

VIII. Division.—*Display of the elementary managemeut of surgical operations.*

*General surgical operations:* Bleeding, cupping, application of issues, introduction of setons, amputations, resections, and so on." 6.

As the author admits, many objections may be made to this arrangement. Thus, under the head " of some peculiar kinds of inflammation," we find " Burns." By a burn is commonly understood an injury to a part by the action of fire; inflammation, common not specific, being generally the consequence, but by no means invariably, for life may be destroyed before time is given for any inflammation to be set up. Again, under the head of " Old solutions which do not suppurate," are placed *hare-lip* and *cleft in the soft palate.* But these malformations are not *solutions* at all. They are the result of an arrest of development; consequently, as a continuity of the parts never existed, the defect cannot properly be termed " *a solution.*" We have remarked other imperfections in the Classification equally striking.

The next Chapter contains a historical sketch of surgery, which is extremely brief and meagre. The author makes a division into five periods.

| | |
|---|---|
| " *First period* . . . | to the time of Hippocrates. |
| *Second period* . . | from Hippocrates to Galen. |
| *Third period* . . | from Galen to the fifteenth century. |
| *Fourth period* . . | the sixteenth century to the middle of the seventeenth. |
| *Fifth period* . . . | the second half of the seventeenth century to the present time." 5. |

The translator has added some particulars in reference to the encouragement given to the pursuit of anatomy in England, which may interest.

" The study of anatomy does not seem to have been so little thought of at this time as generally believed, in proof of which it may be mentioned that Sir Edward Arris, an alderman of London, who was also warden in 1642, and master of the Company of Barbers and Surgeons in 1651, founded on the 27th October, 1645, six anatomical lectures, to be publicly read every year between Michaelmas and Christmas, and endowed them with 300*l.*, on condition that the Company should pay for the lectures 20*l.* a-year: subsequently he exchanged this sum for an annuity of 30*l.* charged on his estates, and at a later period redeemed this charge by paying 510*l.* to the Company, which was by them paid over at the dissolution of the Surgeons' Company, and, when the latter merged into the College of Surgeons, the same was handed over to them. Arris's good example was followed by Mr. John Gale, who, on the 30th June, 1698, founded one anatomy lecture every year, to be called Gale's Anatomy, and endowed it with a rent-charge of 16*l.* a-year out of certain landed property, which was subsequently sold for 432*l.* sterling, and the interest thereon now produces rather more than 20*l.* The two endowments are now consolidated, and the lectures on human Anatomy and Surgery are called Arris's and Gale's Lectures." 8.

After giving a bibliographical account of the literature of surgery, which from its length is calculated as much to frighten as to be useful to students, the author enters upon the first Division, which treats of inflammation. It is stated—

"Inflammation always commences with a more or less intense pain; the sensibility of the part is increased, redness soon follows, and blood appears in vessels where previously it had not been observed; the temperature of the part is raised, its functions disturbed, secretion suppressed, (at least at first,) or changed, perspiration diminished, and the part swelled. These appearances are developed, in different proportions, to a higher degree, in which fever (*Febris inflammatoria secunda)* usually becomes connected with them.

"[I apprehend it would be more correct to say that 'inflammation, from its very commencement, is always accompanied with a more or less intense pain,' than to say, with our author, it 'always commences with a more or less intense pain;' inasmuch as, though that by which the patient's attention is first excited, yet it is only an indication of a disturbance set up in the economy, and which, as it becomes greater, renders itself apparent to the eye, most commonly by redness.]—J. F. S." 24.

After having read, in the preceding paragraph but one, "inflammation is that condition of an organized part in which the vital process and plasticity of the blood are unnaturally raised, and *which is manifested by pain,** redness, increased temperature and swelling," the comment of the translator seems hypercritical.

The following is an excellent description of acute mortification. We know of none superior to it in accuracy and fidelity.

"Mortification truly consists in the extinction of vascular and nervous activity, in consequence of which partial death ensues. This transition is to be feared in unusually severe and quickly developed inflammations with well marked general symptoms in young powerful subjects, and after the operation of severe injuries; in persons with the general appearance of weakness, if the redness of the inflamed part be bluish, of a dirty yellow, the pain slight, and if it be accompanied with typhus. If the pain quickly increase to a great degree, the inflammatory swelling be hard, dry, and very tense, the heat intolerable, the skin dark red, often brownish, the fever extraordinarily severe, and no appearances ensue which lead to the hope of the inflammation terminating in suppuration, then the signs of incipient exhaustion become manifest. The acute pain becomes dull, aching, stretching; there is still indeed circulation, but its current gets slower and slower, and at last stops altogether. The redness therefore becomes deeper, more dusky, and further extended; the warmth diminishes, the swelling, at first hard and tense, becomes soft, doughy, œdematous, the cuticle rises in blisters, containing a dark-coloured brownish fluid. In this condition the part has not yet lost all its sensibility and warmth; the vital activity may therefore be re-awakened and reparation effected. The pulse is small, quick, and loses all fulness and hardness; the patient is depressed, is uneasy, has a languid countenance, cold sweats, dry, dirty tongue, unquenchable thirst, frequently burning hot skin; the features at the same time become pinched, and the urine is thick. When exhaustion of the living activity and fully developed mortification takes place, then the pain ceases entirely, the colour of the part becomes blue, ash gray, or even black, the bone assumes a light white, dirty yellowish, or even black spotted appearance. By the decomposition of the parts still covered with skin, and the evolution of the

* The italics are our own.

gases of mortification an emphysematous swelling is produced, the part becomes quite cold, and the general appearances of exhaustion are present in a higher degree, the mortification either spreads further, and death ensues from exhaustion, or on the confines of the slough is produced a bright redness, suppuration, and by the operation of the absorbing vessels a groove, becoming deeper and deeper, by which the slough is thrown off." 54.

The author has very properly briefly noticed the characters of inflammation as modified in the different tissues—in the skin, cellular tissue, glands, mucous membranes, &c.—in all of which parts he gives a slight sketch of the effects of the disease. We extract the following.

"Inflammation of the coats of *Arteries* is either generally diffused, with violent pulsation of the heart and arteries and high fever; or it is confined to one spot, when the symptoms are commonly obscure. The acute *partial* inflammation of arteries commonly runs into adhesion; the *chronic*, which mostly depends on diseases with little power, into thickening, loosening, ulceration, deposition of calcareous masses, whence commonly results the origin of aneurisms." 74.

To this concise account of arteritis the translator has added notes, closely printed in small type, extending throughout three entire pages, in which he has quoted the observations, valuable we admit, of Bouillaud and Hodgson, and has also minutely detailed a case, furnished him by a friend, in which there is a daily report of the symptoms. The reader will perceive that we were fully justified in our remark that, the author's protracted additions materially alter the character of the original work. This remark equally applies to the next subject, inflammation of the veins, which is dispatched in the text in eight lines, but is followed by more than three pages of additional notes and observations.

On the subject of the treatment of abscess it is stated;—"Only in small abscesses just beneath the skin, and in those in glandular structures may the opening be left to nature."

Mr. South rightly observes—"This is not good practice; abscesses just beneath the skin should always be punctured early, as otherwise there is great risk of sloughing of the integument and the formation of an ugly scar. Neither should abscesses in glands be left to burst, which is often a very tedious process, as the capsule of the gland does not readily ulcerate, and will not till the whole, or nearly the whole, gland is destroyed; a large cavity is thus formed, which is generally very difficult to heal, as it assumes oftentimes a fistulous character. It is therefore always best to puncture a glandular abscess as soon as the capsule and the skin have become adherent, and the angry appearance of the latter indicates its disposition to ulcerate. But it is not unfrequently advisable to open such abscesses before the skin reddens, or even before it is adherent; for, in scrofulous and chronic abscesses, there is often little and sometimes no redness of the skin, and yet, the collection of pus increasing, the skin becomes stretched beyond endurance, and sloughing ensues."

Particular directions are given as to the modes of opening abscesses by cutting instruments, by escharotics and by seton. The two latter are obsolete in this country, and are very properly condemned by the translator, and, as we have already noticed, the necessity for cautions of this nature renders the book less fitted for the hands of students.

The following passage affords a good illustration of the succinct manner in which the author treats practical subjects.

"Gangrene from pressure by lying is to be guarded against by suitable preparation of the bed, by lying on a mattress instead of a feather-bed, by proper cleanliness, frequent change of the body-linen and sheets, repeated alteration of position, by putting doe-skin beneath the patient, by frequent washing the compressed parts with cold water, lead wash and camphorated spirit. If the part have become red, it must be laid on a hollow formed by introduction of ring-shaped pads, little bolsters of horsehair, cleft mattresses, and afterwards compresses moistened with lead wash, vinegar, or Theden's arquebusade water must be applied, or the part must frequently be smeared with an ointment of white of egg and camphor beaten to a cream. When ulcerative absorption has occurred, softening poultices, ointment of oxide of zinc, or of lead, with opium or camphor, should be applied, and, if the ulceration be spreading and deep, aromatic poultices. If actual gangrene be present, then the ordinary treatment for gangrene must be employed. Of course the treatment of the patient's health should be guided by the state of the disease." 96.

The directions here given for obviating the inconvenience and for the treatment of bed-sores would be complete if they included air-cushions and the water-bed. It is probable that Dr. Arnott's valuable invention may not be known in Germany, but we are surprised that Mr. South should have omitted all notice of it. A note mentioning the nature of "Theden's arquebusade water" would also have been useful.

The Chapter on Burns is a good one. Chelius describes four degrees, which we much prefer to Dupuytren's more minute division into six.

"The *first degree* of burn, arising from hot vapour, from the momentary or lengthened touch of a more or less hot body, produces a bright uncircumscribed redness of the skin, as in erysipelas, which for the moment disappears on pressure of the finger, without swelling, and is accompanied only with increased turgescences of the skin and a little pain. Febrile action only sets in if this degree of burn be much spread and in sensitive persons. The redness of the skin either disappears after some hours or days, when the cuticle scales off.

"In the *second degree*, which is most commonly produced by hot fluids, the cuticle rises either at once or gradually into larger or smaller blisters, filled with clear or yellowish fluid, the redness and swelling of the skin is more distinct; the pain severe, burning; and, according to the degree of these appearances and the extent of the burn, do febrile symptoms set in. These blisters either shrivel together and dry, the fluid being absorbed and the skin thrown off, or, if they burst and are opened, the fluid is discharged, the blister falls together, dries, and after some days either a new cuticle is produced or the exposed part suppurates. The healing leaves no scar.

"The *third degree* of burn is usually produced by the flame of fire or by the lengthened touch of hot bodies, especially of hot fluids, and is characterized by gray, yellowish, or brown spots, which are thin and soft, insensible to light pressure, but are painful if the pressure be increased; at the same time generally appear blisters full of brownish or bloody fluid; the surrounding parts are very red and much swollen. The general reaction corresponds to the degree of the inflammation. After six or eight days, and frequently later, the remains of the destroyed cuticle and mucous net are thrown off, and the cure is effected by granulations and the formation of a white glossy scar.

"In the *fourth degree* of burn the destruction penetrates either through the entire thickness of the skin and cellular tissue, or deeper into and through the muscles to the bone, or the whole part is destroyed and charred. This degree is produced by long contact with fire, red hot or molten metals, boiling fluids. The sloughs differ in thickness, are completely insensible; soft, gray, or yellow if produced by hot fluids; brown or black, dry, hard, and sounding when struck, if caused by fire or dry hot bodies. In the immediate neighbourhood of these sloughs the skin is drawn into radiating folds; the surrounding parts are extremely red and swollen, very painful, and frequently beset with blisters. The slough is thrown off by the suppuration which takes place around it, and a more or less deep suppurating space is produced, which commonly has a much larger extent than the slough, because in consequence of the severe inflammation its immediate belt is destroyed by gangrene. The granulations most usually are developed very quickly and luxuriantly, the edges quickly draw together, and shapeless, hard, contracted, tough scars are produced, whereby the direction and motion of the part is often changed and impeded, and the latter even perfectly destroyed. After the throwing off of a part which has been entirely charred, a more or less uneven stump is produced." 111.

The following is an excellent account of the constitutional effects of Burns.

" More or less severe symptoms ensue according to the different degree and extent of the burn, according to the importance of the burnt part, and the constitution of the patient, and not merely does the degree, but also the extent of the burn, determine its danger. In the first two degrees the inflammation is easily resolved, and only if it affects a large extent of the body, and still more in the higher degrees, does febrile reaction set in, when, on account of the disturbed functions of the skin and the changed relations between the external and internal skin, the mucous membrane of the intestines is quickly affected, and uneasiness, loss of sleep, red dry tongue, nausea, vomiting, high nervous excitement, delirium, and the like come on. From the severity of the pain cramps and convulsions occur, especially in sensitive persons. In extensive burns death may ensue rather suddenly from the greatness of the pain, from the quick stopping of the functions of the skin, from the excessive flow of blood to the internal parts where on dissection either no internal derangement is seen, or where a gorging of the brain and mucous membranes with blood and even effusion into their cavities is observed; or from the severity of the fever, especially if accompanied with inflammation of internal parts, of the stomach, intestines, brain, more rarely of the lungs and of the pericardium; or from the very copious and continued suppuration, by which the powers are exhausted. The production of unsightly hard scars, or the growing together of neighbouring parts, may cause disturbance or complete stoppage of their functions." 111.

In his usual concise manner the author has dismissed the subject of the Treatment of Burns in little more than half a page. The translator, however, compensates for this brevity by giving eleven pages of additional notes, fully detailing the views of Kentish and many others on the treatment of these injuries. We fully agree with Mr. South in the following remarks.

" It is matter of dispute among surgeons, as to the propriety of administering opiates or other sedatives, an objection being made that the action of the opium interferes with the symptoms, so that it is impossible to determine whether the brain be affected by the irritation of the injury or by the action of the opium. I do not think this is matter of much consequence; but I am quite certain that soothing the patient's sufferings and dulling his nervous irritability, are most important indications in the constitutional treatment. Therefore I invariably prescribe laudanum for an adult or syrup of poppies for a child, if there be any disposition to restlessness; and, if it continue, the opiate is repeated a few hours after. The advantage derived from sleep, even for a short period, is very great, and cannot be too strongly recommended, as during that time the stinging of the burn diminishes, and the patient suffers less. If sleep at night be deficient or much disturbed, I think it better to give laudanum in sufficient quantity to procure it; in less quantity it irritates and is hurtful, and this may be continued for some time, as may be found necessary." 120.

We consider it an omission that the translator has neglected to notice the acute ulcer of the duodenum which Mr. Curling has shown to be of common occurrence after severe burns, and is often the immediate cause of a fatal termination by causing diarrhœa or hæmorrhage into the bowels. Long notes are given on the subject of scars, and the treatment of contractions after the healing of burns, and the translator gives the minute particulars of a case operated on by him according to Earle's method, but in which he admits that success was by no means decisive, though the case was proceeding satisfactorily.

In the chapters on Frostbite, Boil, and Carbuncle, we find nothing remarkable.

The third section treats of Inflammation in certain special organs. We think the arrangement by which inflammation of the various organs is described separately from the other affections of the part, a faulty one. This head embraces so many subjects, that we can only find space to allude to a few points of novelty and interest. Women who do not suckle, or who wean their children early, are recommended, in order to prevent inflammation of the breast, to "use a strict diet, encourage perspiration, take purgatives; *apply cotton fumigated with sugar to the breasts, rub the nipples often with spittle,* and support the breasts." The directions given by us in italics, our readers will think better suited for old-fashioned nurses than for the students of 1845. In reference to the disputed point of practice, the opening of breast-abscess, we entirely concur in the following views which are well-expressed by Mr. South.

"Chelius's recommendation of leaving 'the opening of the abscess to Nature' must on no account be followed, as its certain result is, according to his own observation, the occurrence of 'many openings at different parts' of the breast, and the necessary production of very unsightly scars, which most grievously annoy the patient and her friends, and deservedly discredit the reputation of the medical attendant. The abscess is *always to be punctured freely, so soon as fluctuation can be distinctly felt,* and whilst the walls of the abscess are still thick. The almost immediate ease gained by relieving the tension of the fibrous covering of the breast gland is the first advantage obtained; the burrowing of pus is also prevented, and thereby a smaller cavity left, when emptied, to fall together and fill up by granulation; and, most important of all, the sloughing of the skin almost to a certainty precluded. No squeezing or kneading of the breast to evacuate the pus, as often most improperly practised, is to be on any account resorted to, the agony thereby produced is extreme, the benefit gained nothing, for the aperture made should be sufficiently large to permit the free escape of the matter, which, having been allowed to flow as long as it will, a strip of lint, oiled, is to be introduced between the lips of the wound to prevent their union, and a light bread poultice, or warm fomenting flannels laid over the breast and repeatedly renewed. In the course of a few hours the lint should be withdrawn, and the wound generally remains sufficiently open to permit the continual flow of the pus. If, as not unfrequently happens, clots of adhesive matter, or dead cellular tissue, block up the opening so that the matter does not readily escape, they may be gently removed if they protrude between the lips of the wound. But, if not, and the pus be still retained, a grooved director should be very gently introduced into the cavity of the abscess, and by its canal the discharge will pass; but no pressure is on any account to be used. If a second, or even a third, abscess point, or if the same abscess point at a different part of the breast, these are severally to be opened as they occur, the prime object of the treatment being to remove every chance of sloughing and scar of the skin. Oftentimes the first discharge is extremely fetid, more particularly if the opening of the abscess have been delayed, or if it have been left to burst spontaneously, and in these cases the constitutional excitement is frequently very great, amounting even to delirium. The character of the suppuration, however, usually soon becomes healthy, and the febrile symptoms speedily subside. Chelius's objections to passing tents or tubes into the fistulous passages, which generally alone occur from leaving the abscess to burst of itself, are well founded; they never ought to be employed. Neither should Langenbeck's plan of introducing ligatures be for a moment thought of; it is very bad practice.

"Fistulous passages almost invariably occur from the pus not having a convenient and complete discharge. Sometimes gentle, well-applied pressure along the course of the sinuous passage may be sufficient to produce inflammation and adhesion of its walls; but, if not, or if the patient cannot, as sometimes happens, bear the necessary pressure, then a probe should be introduced, and its extremity cut down upon through the skin at that part of the sinus which is most depending. Usually in a few days the old aperture heals, the pus is discharged by the new wound, and soon a cure is effected. As a general rule, injections of these adventitious canals are not advisable; but, when the opening is at the most depending part, and they can be employed simply to wash out the canal and slightly irritate it, but without being retained, which will often create more inconvenience than that to be got rid of, then they may be used with discretion. A mild solution of sulphate of zinc is, I believe, the best injection." 152.

In the notes appended to the chapter on Inflammation of the Urethra, the translator observes, "I once saw a case, under my colleague Mackmurdo's care, in which there was enormous extravasation of blood, from bursting of some vessel in the penis, during the act of coition; and the result of which was, the penis especially and the perinæum were greatly distended, and he was unable to pass his urine without extreme pain, in consequence of which a catheter was introduced. In the course of two or three days, extravasation of urine ensued, and the bladder was punctured through the rectum. Considerable sloughing, not only in the perinæum, but also up into the groins, took place, into which incisions were made, as needed, and he ultimately, though slowly, recovered."

Cases of obstinate erection from extravasation of blood in the corpus spongiosum occurring under the violent excitement of coition have not been much noticed by writers. We have witnessed some cases of it, and have found the priapism subside gradually as the blood became absorbed. We recollect some time back reading a case, in which a surgeon thought it necessary to cut into the corpus spongiosum to relieve the extreme tension of the penis, and the patient ever afterwards lost the power of erection.

On the subject of gonorrhœa, we observe many erroneous views in the text, which the translator does not omit to correct in his notes. We suppose few of our friends have met with the following affection.

"*Gonorrhœa of the Nose* sometimes occurs during gonorrhœa of the urethra, or whilst there is an enlargement of the testicle from the same cause. The

Schneiderian membrane is tender over its whole surface, but not painful; is of a deep red colour, but not ulcerated; and there is a free discharge similar to that of clap.

"This disease was first noticed by Benjamin Bell, and he mentions two cases of it: in the first, 'the discharge from the urethra lessened before the testes became inflamed, and, on this taking place from the nose, it ceased entirely.' It was treated with an astringent lotion and the insertion of sponge moistened in it up the nose, and cured in a few days. In the second case, 'the discharge took place during the continuance, and had existed many years, and, although it had frequently become less, it never disappeared entirely.' Various attempts at its cure were made without success, 'and, though no other symptom appeared, he was advised to undergo a course of mercury; but no advantage ensued.' (pp. 29, 30.)—J. F. S." 177.

In treating of Inflammation of the Testicle, the author states that it may be a symptom of general syphilis, but he draws no clear distinction between the ordinary form of acute orchitis, and the peculiar chronic inflammatory enlargement which occurs as a secondary, or rather tertiary, symptom of the venereal disease. After giving the opinions of Sir Astley Cooper and Ricord, in addition to that of Chelius, respecting this affection, we are surprised to find the translator remark, "notwithstanding these high authorities, I must confess I have great doubt as to the swelling of the testicle depending on a syphilitic cause." We thought that the existence of this form of the disease was a fact in pathology too well established to admit of being questioned.

We observe many obsolete opinions and objectionable remarks in this Chapter. Take for instance the following—"Not unfrequently, as a symptom of general venereal disease, a hard swelling of the testicle is slowly produced, which may generally be dispersed by a regular mercurial treatment, or by the proper exhibition of cubebs." It is too obvious that the author confounds the two distinct affections, gonorrhœal and syphilitic orchitis. Again, it is stated, "The hardening of the testicle from one gonorrhœa is not unfrequently dispersed by a fresh gonorrhœa; hence the proposition of passing bougies smeared with red precipitate ointment (not with gonorrhœal mucus) to excite fresh inflammation of the urethra." We have here both bad pathology and bad practice passed over without comment from the translator.

The heading of the next Chapter is "Inflammation of the Lumbar Muscles," and our readers will be surprised to learn that, under this title, a description is given of Psoas or Lumbar Abscess. The history of the disease given by Chelius is extremely meagre, but Mr. South makes amends for the imperfect description of the author by collating the views of the best writers on the affection.

Inflammation of the nail-joint, or Whitlow, occupies thirteen pages of the work, but we find nothing particularly calling for remark.

We here conclude our review of the two first parts of Chelius's System of Surgery. The extracts which we have given will enable our readers to form an opinion of the character of the work. The original German is rendered in good readable English, and much pains have been taken by Mr. South in collating the views and observations of the best writers on the subjects imperfectly treated of in the text. And although we have not been able to give our unqualified approbation to the work, we can recommend it as containing a large amount of valuable information on the most important subjects of surgery.

We will conclude with a hint, which those interested in the work will find well worthy of attention. Be punctual in the issue of the parts at the periods promised in the advertisement. There have been of late such flagrant breaches of faith in respect to books published in parts, that the public are naturally suspicious of them, and disinclined to make an investment they may have good reason to repent.

